# Circulating miR-26a as Potential Prognostic Biomarkers in Pediatric Rhabdomyosarcoma

**DOI:** 10.3389/fgene.2020.606274

**Published:** 2020-12-10

**Authors:** Lucia Tombolan, Caterina Millino, Beniamina Pacchioni, Manuela Cattelan, Angelica Zin, Paolo Bonvini, Gianni Bisogno

**Affiliations:** ^1^Institute of Pediatric Research (IRP), Fondazione Città della Speranza, Padua, Italy; ^2^Functional Genomics Laboratory, Department of Biology, University of Padua, Padua, Italy; ^3^Department of Statistical Sciences, University of Padua, Padua, Italy; ^4^Department of Woman’s and Children’s Health, Hematology and Oncology Unit, University of Padua, Padua, Italy

**Keywords:** miR-26a, circulating microRNAs, ddPCR, pediatric tumor, rhabdomyosarcoma

## Abstract

Rhabdomyosarcoma (RMS) arises from myogenic precursors that fail to complete muscle differentiation and represents the most frequent soft tissue sarcoma in children. Two major histological subtypes are recognized: alveolar RMS, characterized by a more aggressive behavior and a greater proneness to metastasis, and embryonal RMS which accounts for the 80% of cases and carries a better prognosis. Despite the survival of patients with localized tumors has progressively improved, RMS remains a challenging disease especially for metastatic patients and in case of progressive or recurrent disease after front-line therapy. MicroRNAs, a class of small non-coding RNA, have emerged as crucial players in cancer development and progression, and their detection in plasma (circulating miRNAs) represents a promising minimally invasive approach that deserve to be exploited in clinical practice. We evaluated the utility of circulating miRNAs as diagnostic and prognostic biomarkers in children with RMS profiling miRNAs from plasma of a small cohort of RMS patients and healthy donors (HD) using a qPCR Cancer Panel. An assessment of hemolysis status of plasma using miR-451/miR-23a ratio was performed as pre-analytical analysis. Statistical analysis revealed that miRNAs expression pattern clearly distinguished RMS patients from HD (*p* < 0.05). Interestingly, plasma levels of muscle-specific miR-206 were found to be significantly increased in RMS patients compared to HD, whereas levels of three potential tumor-suppressor miRNAs, miR-26a and miR-30b/30c, were found lower. Reduced levels of circulating miR-26a and miR-30b/c were further measured in an independent larger cohort of patients (validation set) by digital droplet PCR. In particular, we evidenced that miR-26a absolute plasma levels were associated with fusion status and adverse outcome (*p* < 0.05). Taken together, these findings demonstrate the potential of circulating miRNA as diagnostic and prognostic biomarker in children affected by this malignancy and enforced the key role of miR-26a in pediatric rhabdomyosarcoma.

## Introduction

Rhabdomyosarcoma (RMS) is the most common soft tissue sarcoma affecting children. It arises from myogenic precursor that are unable to complete muscle differentiation. Typical myogenic factors such as MyoD1 and myogenin are expressed by tumor cells and routinely used to define the diagnosis of RMS (Sebire and Malone, 2003). Two major histotypes are recognized: alveolar RMS (ARMS) and embryonal RMS (ERMS). About 80% of ARMS are characterized by a reciprocal translocation, the more frequent t (2;13) and the less common t (1;13) (Rudzinski et al., 2015). Both translocations involve PAX genes, PAX3 and PAX7 respectively, and the transcription factor FOXO1. Fusion-positive ARMS tumors are associated with a more aggressive phenotype and worse prognosis respect to the embryonal RMS(Sorensen et al., 2002). Despite the survival of RMS patients remarkably increased during the last years thanks to the adoption of a multidisciplinary treatment modalities, patients presenting metastatic disease at diagnosis or experiencing relapse after treatment still have a dismal prognosis, with chances to reach a long survival lower than 5% (Oberlin et al., 2012). Currently, detection of relapse in children with RMS includes bone marrow aspirate and biopsy and computed tomography (CT) scan (Huh and Skapek, 2010).

Herein, the detection of specific biomarkers in peripheral blood or other biological fluids may help to predict the probability of a relapse or a progressive disease in pediatric RMS.

The identification of biomarkers in biological fluids termed as liquid biopsy is a very promising field of investigation in solid tumors. Liquid biopsy is a minimally invasive approach with the potential of providing information on early diagnosis, prognosis and to monitor the response to treatment.

Circulating microRNAs (miRNAs) are endogenous non-coding RNA that have also been detected in many biological fluids, such as urine and plasma/serum.

MiRNAs can be released in the circulation as both cell-free miRNAs and packed into a secretory vesicles known as exosomes. Cell-free miRNAs are complex to proteins such as argonaute proteins (AGOs) or high density lipoproteins that confer to them an extremely stable form resistant to ribonuclease activity (Mitchell et al., 2008; Arroyo et al., 2011; Vickers et al., 2011).

MicroRNAs are able to finely modulate the expression of their target genes through a specific and well known mechanism which leads to target mRNA degradation or inhibition of protein translation (Bartel, 2004). MicroRNAs are involved in many physiological and pathological conditions that include tissue development and tumor-related processes. In RMS, several functional studies demonstrated that specific miRNAs can act both as tumor suppressor and oncomiRs thus regulating proliferation, invasion and apoptosis of cancer cells (Tombolan et al., 2015; Molist et al., 2020; Wang et al., 2020).

Numerous studies have demonstrated that miRNAs expression profiling varies between tumor and normal tissues as well as different tumors, like sarcomas, display a peculiar miRNAs expression pattern that contributes to define tumor phenotype (Varshney and Subramanian, 2015; Smolle et al., 2017).

MiRNAs play also a crucial role in skeletal muscle development in which tissue-specific miRNAs, called myomiRs (miR-1, miR133a, miR-133b, and miR-206) tightly regulate the differentiation and proliferation of myogenic precursor cells (McCarthy, 2008; Horak et al., 2016). Interestingly, in RMS myomiRs, that result significantly downregulated, act in concert with other non-muscle miRNAs (miR-29, miR-26a, and miR-183) impairing myogenesis and proliferation. MyomiRs in RMS interact directly with PAX3 and PAX7 genes as well as with cell cycle regulator Cyclin D2 and cMET oncogene (Rota et al., 2011; Li et al., 2012; Gasparini et al., 2019).

In this study, we investigated the expression of miRNAs in the plasma of two cohorts of RMS patients and healthy subjects to figure out whether miRNAs represent useful diagnostic and prognostic biomarkers in pediatric rhabdomyosarcoma. We used qPCR to test a panel of about 84 miRNAs in a small training group of patients and controls then we verified the expression of three miRNAs in a validation cohort of samples taking advantage of the droplets digital PCR (ddPCR) technique. Our findings unveiled that circulating miRNAs can distinguish RMS patients from healthy subjects and, as for miR-26a, can correlate with an increased risk of relapse and a poor prognosis.

## Materials and Methods

### Blood Samples and Ethical Issue

A total of 30 blood samples from RMS patients enrolled in national and international pediatric sarcoma protocol (EpSSG RMS 2005) were analyzed in this study: 8 were processed with qPCR Cancer Panel (training group) and 22 with droplet digital PCR (validation group). All the clinical features are summarized in Table 1. In addition, 8 healthy donors were included in the study as controls. Peripheral blood of RMS patients was collected at the time of diagnosis, prior to any treatment, in sodium citrate tubes and processed within 24 hours. Plasma was obtained from peripheral blood after two steps of centrifugation: the first at 890 × *g* for 10 min and the second at high speed (16000 × *g* for 10 min) then the samples were stored at −80°C in the Pediatric-Oncology biobank (BBOP) (part of Italian Association of Pediatric Hematology and Oncology (AIEOP). This study was approved by Padua Hospital Ethics Committee (No. 988P, 31March 2005) and the patients have signed informed consent.

**TABLE 1:** Clinical features of RMS patients analyzed. GUBP = GenitoUrinary Bladder-Prostate, GUnoBP = GenitoUrinary non-Bladder-Prostate, HNnoPM = Haed Neck non-Parameningeal, HNPM = Haed Neck Parameningeal; IRS group, Intergroup Rhabdomyosarcoma Studies (Clinical Group), n.a. = data not available.

Variable	RMS (training set)	RMS (validation set)
**Histotype**		
Alveolar	4	10
Embryonal	4	10
Other	0	2
**Fusion Status**		
PAX3/FOXO1	4	6
PAX7/FOXO1	0	3
NONE	4	13
**Age, years**		
≤10	4	13
>10	4	9
**Sex**		
Male	3	14
Female	5	8
**Site of disease**		
GUBP	1	0
GUnoBP	0	2
HNnoPM	0	1
HNPM	2	9
Limbs	0	5
Orbit	1	1
Other sites	4	4
**Size**		
≤5 cm	2	6
>5 cm	6	14
n.a.	0	2
**IRS group**		
I	0	0
II	0	0
III	5	14
IV	3	7
n.a.	0	1
**Event**		
Yes	4	11
No	4	11
**Status of disease**		
Dead	5	11
Alive	3	11

**TABLE 2:** Comparison between miRNAs datasets.

13 common elements	Missiaglia et al., 2017 “RMS vs. sk ms” Sample type: Tissue				Tombolan et al., 2020 “RMS vs. HD” Sample type: Plasma		
		
ID	logFC	AveExpr	*P*-Value	FDR	FC	log FC	*P*-value
**hsa-let-7d-5p**	**−0,32**	4,37	0,00621581	0,02569494	−5,34	**−2,42**	0,00088406
**hsa-miR-181b-5p**	0,39	4,11	0,0019584	0,01013688	−2,10	−1,07	0,01209047
**hsa-miR-191-5p**	**−0,31**	3,54	0,00732697	0,0291309	−3,81	**−1,93**	0,00937036
**hsa-miR-19a-3p**	**1,01**	3,72	2,5271E-09	3,7729E-07	2,34	**1,23**	0,0003896
**hsa-miR-19b-3p**	**0,48**	3,98	0,0002534	0,00200527	1,74	**0,80**	0,00608738
**hsa-miR-206**	−0,50	4,62	0,00054228	0,00362842	108,93	6,77	0,00051132
**hsa-miR-23a-3p**	**−0,45**	4,13	0,00029463	0,00228758	−1,91	**−0,93**	0,00460076
**hsa-miR-23b-3p**	**−0,84**	3,79	2,0847E-07	7,7988E-06	−1,91	**−0,93**	0,01025567
**hsa-miR-26a-5p**	**−0,48**	4,29	6,2407E-05	0,00068482	−4,49	**−2,17**	0,00399244
**hsa-miR-29a-3p**	−1,27	3,34	9,9963E-08	4,33E-06	2,06	1,04	0,0072167
**hsa-miR-29c-3p**	−1,38	3,17	8,7252E-13	7,1808E-10	2,39	1,26	0,00454803
**hsa-miR-30b-5p**	**−0,61**	3,67	4,8404E-05	0,00055329	−3,66	**−1,87**	0,00268519
**hsa-miR-30c-5p**	**−0,71**	3,78	1,2507E-05	0,00018715	−5,91	**−2,56**	3,8173E-05

*Matching of circulating miRNAs differently expressed in RMS and HD (right panel) plasma samples (Tombolan et al., right) and in RMS tumors and skeletal muscle cells (Missiaglia et al., left). Almost 50% (13 out of 30) of circulating miRNAs identified by the qPCR Cancer Panel assay were also found deregulated in RMS tumors. miR26a-5p and miR30b/30c showed comparable fold change values in the two datasets, whereas MiR-206, miR-29a/c, which are muscle-specific miRNAs, are deregulated in skeletal muscle cells (Missiaglia dataset) but up-regulated in RMS patients’ plasma compared to healthy donor’s plasma samples (qPCR Cancer Panel). In bold miRNAs with a concordant expression. logFC: log2 of the fold change; AveExp: average log2-expression; FDR: false discovery rate.*

### Extraction of Circulating miRNAs From Plasma

Before starting the miRNA extraction procedure, plasma samples were centrifuged at 3,000 × *g* for 5 min to remove all cellular debris. Then, 1 μl of Exiqon RNA spike-in mix (UniSp2, UniSp4, and UniSp5) and 2 μl 1 μM of ATH-miRNA159a were added to 200 μl of plasma (starting volume) to control the sample-to-sample variation in the RNA isolation procedure. Subsequently, isolation of plasma miRNAs was performed according to the manufacturer’s instructions of miRCURY RNA Isolation kit Exiqon.

### Assessment of Hemolysis

Hemolysis in plasma samples was measured by two different methods. The first method uses a NanoDrop spectrophotometer (Thermo Fisher Scientific, Waltham, MA, United States) to measure the absorbance of hemoglobin at 414 nm (2 technical replicates per samples was performed). The second method is based on qPCR analysis of miRNA-451 and miRNA-23a-3p. A ratio between two miRNAs calculated as delta Cq (miRNA-23a-3p – miRNA-451) was used as hemolysis indicator. A ratio more than five indicates a high risk of hemolysis (Blondal et al., 2013). It was estimated that absorbance method has a hemolysis limit of detection of 0.004% while miRNA ratio has a limit of 0.001% (Shah et al., 2016).

### Reverse Transcription and Quantitative PCR (qPCR)

cDNA was synthesized using a miRCURY LNA Universal cDNA Synthesis Kit II (Exiqon) starting from 6 μl of total RNA with the addition of 1 μl of UniSp6 and 1 μl of Cel-miR-39 as exogenous miRNA spiked-in control. The RT product was used as a template in the qPCR assays in Ready-to-use Human Cancer Focus microRNA PCR Panel V4 (Supplementary Table S1, Exiqon) and ExiLENT SYBR Green master mix (Exiqon). ROX (Invitrogen by Life Technologies, Carlsbad, CA, United States) was added to the master mix as the passive reference dye. The arrays were run in an ABI7500 Real-Time PCR System (Applied Biosystems, Foster City, CA, United States). The qPCR cycling conditions were 95°C for 10 min and 40 cycles (95°C for 10 s and 60°C for 1 min).

### Droplets Digital PCR (ddPCR)

For each ddPCR assay, 9 μl cDNA sample (diluted 1:20), 10 μl 2X ddPCR supermix for Eva Green (Bio-Rad, Hercules, CA, United States), 1 μl of LNA PCR primers set was mixed (miRCURY LNA miRNAs PCR assays, Qiagen, Hilden, Germany). The mixture and 70 μl of droplet generation oil for Eva Green were respectively loaded into the sample and oil wells of a disposable droplet generator cartridge (Bio-Rad, Hercules, CA, United States). Therefore, droplets were generated by QX200 droplet generator device (Bio-Rad) and transferred to a 96 plate (Bio-Rad, Hercules, CA, United States). The cycling condition were 95°C for 5 min and 40 cycles (95°C for 30 s 58°C for 1 min) 4°C for 5 min and 90°C for 5 min. At the end of the PCR reaction, droplets were loaded in the QX200 droplet reader and analyzed using Quantasoft version 1.7.4 software (Bio-Rad, Hercules, CA, United States). A no template control (NTC) was included in every assay. MiRCURY LNA miRNAs PCR assays used are: miRNA-26a, miRNA-26b, miRNA-30b, miRNA-30c (Qiagen, Hilden, Germany).

### Statistical Analysis

The Human Cancer Focus microRNA PCR Panel V4 (Exiqon) data analysis was carried out using GenEx Enterprise software (Exiqon). Both data exported form ABI7500 and the analysis were performed following the instruction reported in GenEx Enterprise software manual. In details, first we performed a pre-processing of the data that include an interpolate calibration using the spike UniSp3, a cut-off setting to exclude data with very high Ct (threshold cycle > 37), an estimation of frequency of missing data and a log conversion of the values. Then data were normalized using different methods: 1. Using 5 different reference miRNAs chosen based on the lowest standard deviation between samples; 2. Using the global mean normalization, a method often applied to gene expression data. Statistical analysis was performed on normalized data to compare RMS vs. healthy donors (HD) (*t*-test) or to compare 3 different groups: Alveolar (A), embryonal (E) and HD (ANOVA). The *p*-value (*p* < 0.05) was calculate. Cluster analysis was performed using morpheus software^1^, Broad Institute) while principal component analysis (PCA) plot was created with BioVinci software (BioTuring Inc., San Diego, CA, United States).

Droplet digital PCR data were analyzed using GraphPad Prism 6 and R statistical software. The association of miR-26a, miR-26b, miR30b, and miR-30c with clinical features were performed using non-parametric Mann-Whitney and Kruskal-Wallis tests. All *p*-values were considered statistically significant at the alpha level of 0.05. Kaplan–Meier survival curves were analyzed with log-rank test to estimate the prognostic value of miR-26a expression.

## Results

### Circulating miRNAs Expression Profiles Distinguish RMS Patients From Healthy Donors

With the aim to explore the circulating miRNAs RMS profile in patients’ plasma samples we took advantage of a qPCR panel of 84 cancer-associated miRNAs (*Cancer Panel*). Starting from a small volume of plasma (200 μl) we initially tested the hemolysis status of each sample by using the miR-451/miR-23a ratio as described in the method section. It is known that hemolysis grade affects miRNAs plasma levels so this pre-analytical analysis is mandatory (Blondal et al., 2013; Shah et al., 2016). Only the samples that passed the hemolysis test were used in the following experiments (see Supplementary Table S2). We applied the qPCR Cancer Panel to a training group of plasma samples composed of 8 RMS patients (4 PAX3/FOXO1-positive ARMS and 4 fusion-negative embryonal RMS, ERMS) and 4 healthy donors (HD, *controls*), and quantified the differential expression of the 84-cancer associated miRNAs as reported in the Supplementary Table S3. Data normalization was performed applying two different methods: global mean (GB) and 5 reference genes (5RG) normalization. The Volcano plot displayed a good distribution of normalized data using either one of the two normalization approaches (see Supplementary Figures 1A,B), and when unsupervised hierarchical clustering analysis was performed using all miRNAs of the qPCR Cancer Panel a good separation between groups (ARMS, ERMS, and HD) was observed (Supplementary Figure 1C).

**FIGURE 1:**
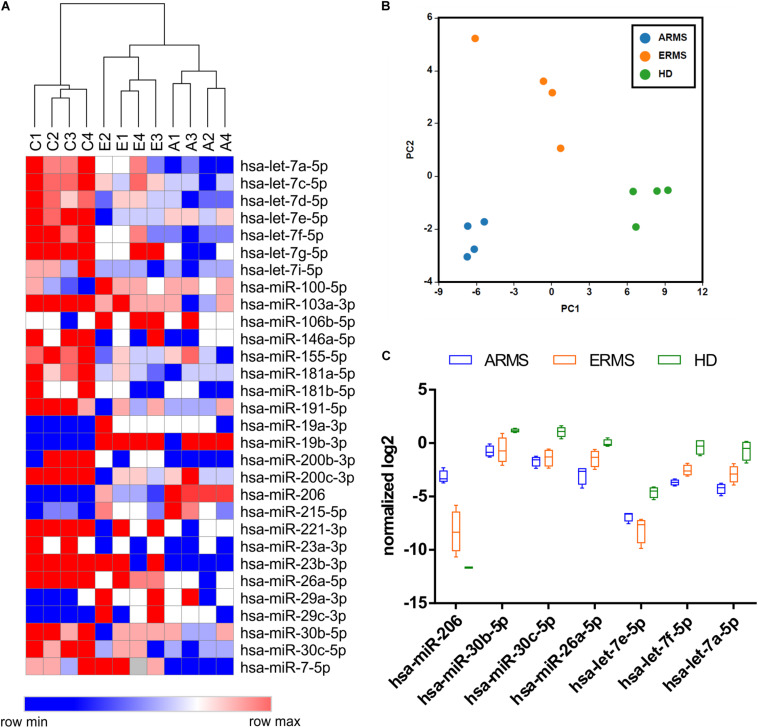
Circulating miRNAs expression profiles distinguish RMS patients from healthy donors. **(A)** The heatmap shows the circulating miRNAs expression profiles in 8 RMS samples (4 ERMS and 4 ARMS PAX3/FOXO1) and 4 healthy donors based on hierarchical clustering. The statistically different miRNAs were obtained using both global mean and 5 reference genes normalization. **(B)** The Principal component analysis (PCA) shows the clear separation of three groups (HD, ERMS, and ARMS) based on circulating miRNAs expression patterns. **(C)** Box plots of relevant miRNAs deregulated in RMS respect to HD are reported. C1–4 = healthy donors; A1–4 = alveolar RMS; E1–4 = embryonal RMS.

We identified a set of miRNAs differentially expressed between RMS patients and healthy donors with both normalization approaches, and observed a high percentage of common deregulated miRNAs (about 80%) between the two analysis, consistent with the high confidence of the data obtained (see Supplementary Figure 1D). The hierarchical clustering analysis performed using the differentially expressed miRNA demonstrated a clear separation between RMS cases and controls, as well as between alveolar and embryonal RMS patients (*p*-value < 0.05) (Figure 1A). The list of miRNAs differentially expressed was reported in Supplementary Table S4. Similarly, the Principal Component Analysis (PCA) confirmed the presence of distinct groups of samples with embryonal group more similar to controls with respect of alveolar ones (Figure 1B).

We, then, focused our attention on the miRNAs significantly different between RMS patients and HD. As expected, miR-206, a known muscle-related miRNA extensively studied in RMS, displayed higher levels in RMS plasma samples, especially in the alveolar subtype, respect to controls, whereas, like some members of the let-7 family, miR-26a and miR30b/30c, whose tumor suppressor role in other cancers has been described, were downregulated in RMS plasma samples compared to controls (Figure 1C). We also compared our results with a previously published dataset by Missiaglia et al. (2017) obtained analyzing the miRNAs profiling of 64 RMS samples by next-generation sequencing. Although the miRNAs profiling conducted by Missiaglia and colleagues was performed on tumor tissues and skeletal muscle cells as the normal counterpart, we observed that almost 50% (13 out of 30) of circulating miRNAs deregulated in plasma of RMS patients with respect to healthy donors was also deregulated in primary RMS tumors. Accordingly, miR-26a and miR30b/30c were downregulated in RMS samples of both datasets, whereas miR-206 and miR-29b/c expression levels were apparently in contrast. However, since it is known that both miR-206 and miR-29b/c are involved in myogenesis (Wang et al., 2008; Horak et al., 2016) their downregulation in differentiated skeletal muscles and overexpression in RMS plasma sample is consistent and reasonable.

### Validation of Selected miRNAs in an Independent Cohort of RMS Patients Using ddPCR

Droplet digital PCR (ddPCR) is a relatively recent technique providing equal results to qPCR with the advantage to perform an absolute quantification without the need of normalization with internal/external references. Among its applications, the quantification of circulating miRNAs in blood is very promising.

**FIGURE 2:**
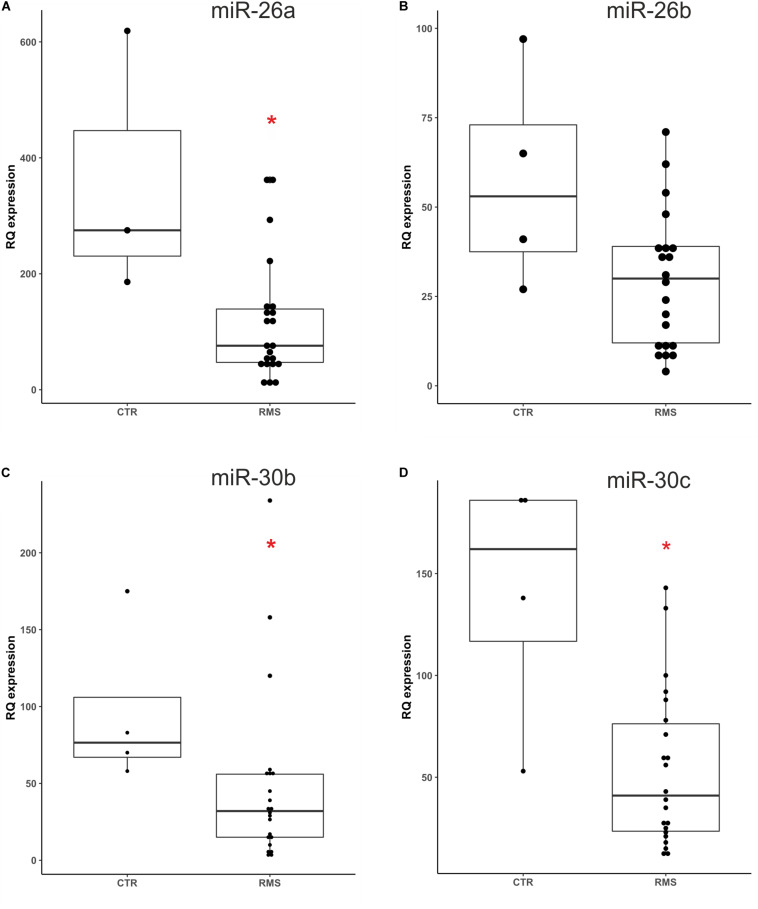
Absolute quantification of miR26a/b and miR-30b/c plasma levels in an independent cohort of RMS patients. The expression levels of four selected miRNAs were assessed by ddPCR in a novel group of RMS patients and healthy donors. Mann-Whitney test sustained miR-26a **(A)**, miR30b **(C)**, and miR-30c **(D)** different plasma levels in RMS patients compared to controls (CTR), whereas miR26b levels **(B)** resulted not statistically significant (miR-26a *p* = 0.0329*, miR-30b *p* = 0.0209*, miR-30c *p* = 0.0209*, and miR-26b *p* = 0.0947 n.s). *p < 0.05.

Based on literature data we focused our study on three differentially expressed miRNAs, miR-26a, miR-30b and miR-30c that have a potential role as tumor suppressor. We also included in this analysis miR-26b which belongs to the same miRNA family, but was not found downregulated as miR-26a. We first optimized the ddPCR conditions such as primers annealing temperature and template dilution for each assay. We performed ddPCR on 26 plasma samples (22 RMS and 4 healthy donors) used as validation group of the data obtained with the qPCR miRNAs Cancer Panel described above. The clinical characteristics of the RMS patients studied are summarized in Table 1. Analyzing ddPCR data (expressed as copies of target/μl) of each miRNA we validated the different expression of miR-26a miR-30b/30c between RMS patients and controls, while miR-26b was not statistically different between the two groups (Figure 2, miR-26a *p* = 0.0329; miR-30b *p* = 0.0209; miR-30c *p* = 0.0209; miR-26b *p* = 0.0947, Mann-Whitney test). Therefore, we validated with an independent technique and in a novel cohort of patients the data obtained with qPCR Cancer Panel used as a discovery tool.

### Correlation of miR26a and miR30b/30c Plasma Levels and RMS Prognostic Factors

To investigate any correlation between miR26a and miR30b/30c plasma levels and known RMS prognostic factors we performed an association analysis including histology, fusion status, tumor size and clinical stage (according to IRS grouping) RMS clinical features.

**FIGURE 3:**
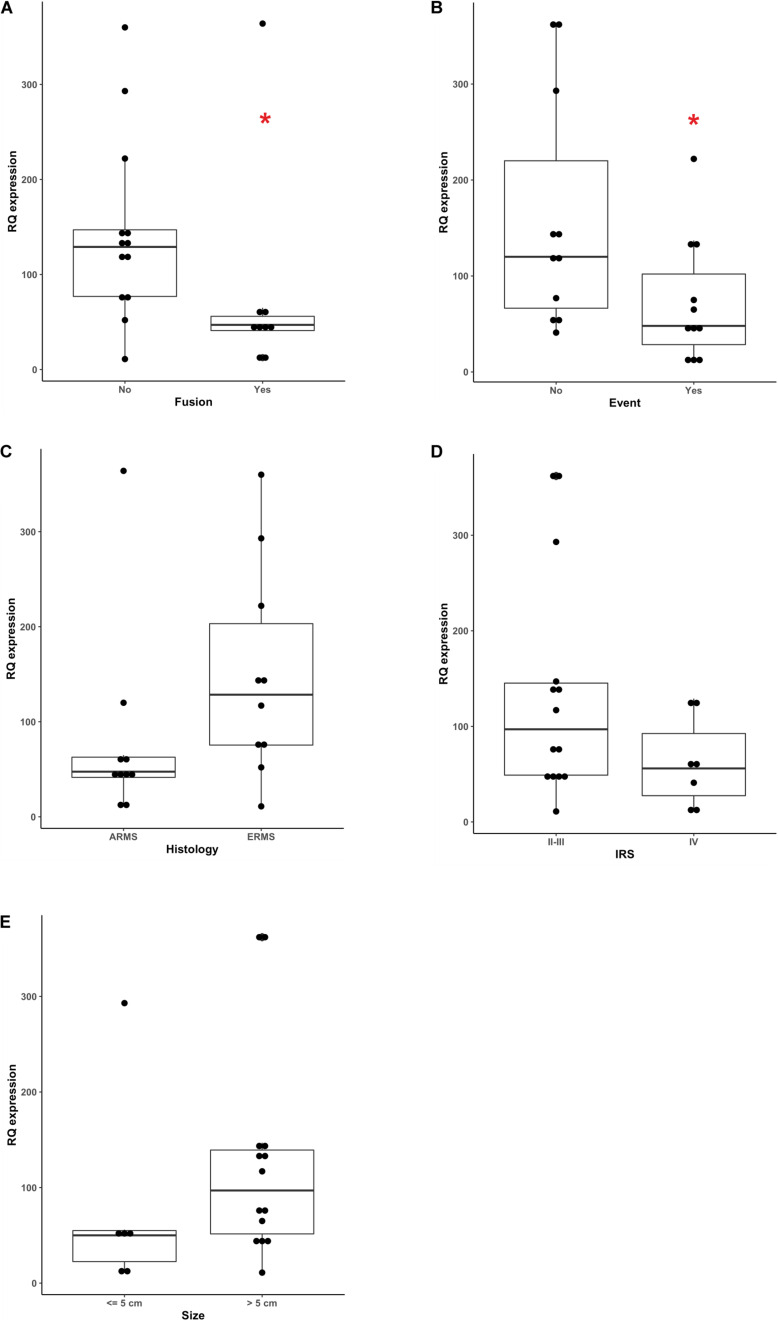
Correlation between miR-26a plasma levels and clinical prognostic factors. miR-26a levels assessed by ddPCR showed significant correlation with, **(A)** fusion status (*p* = 0.0177), **(B)** the occurrence of an event during the follow-up (*p* = 0.417) while no correlation was observed with **(C)** histology (*p* = 0.0587) **(D)** IRS group factors and **(E)** tumor size. Mann-Whitney test was used as non-parametric statistical test (*p < 0.05).

We demonstrated that circulating miR-26a expression inversely correlated with RMS fusion status (*p* = 0.0177, Mann-Whitney test), since PAX3-7/FOXO1-positive RMS cases displayed lower levels of miR-26a compared to fusion-negative ones (Figure 3A), as it was in ARMS (mostly fusion-positive) than ERMS patients (all fusion-negative), although such a difference was not statistically significant (*p* = 0.0587 Mann-Whitney Figure 3C). When event occurrence (local relapse or distant metastasis at follow up) and circulating miR-26a levels were confronted, low levels of miR-26a characterized significantly RMS patients with progressive disease (*p* = 0.0417 Mann-Whitney Figure 3B), whereas clinical risk group (IRS group) and tumor size were not associated with circulating miR-26a expression (Figures 3D,E). Finally, no significant association was found between miR30b/30c plasma levels and RMS prognostic factors (Supplementary Figures 2, 3).

### miR-26a Low Levels Correlate With an Increased Risk of Adverse Outcome

**FIGURE 4:**
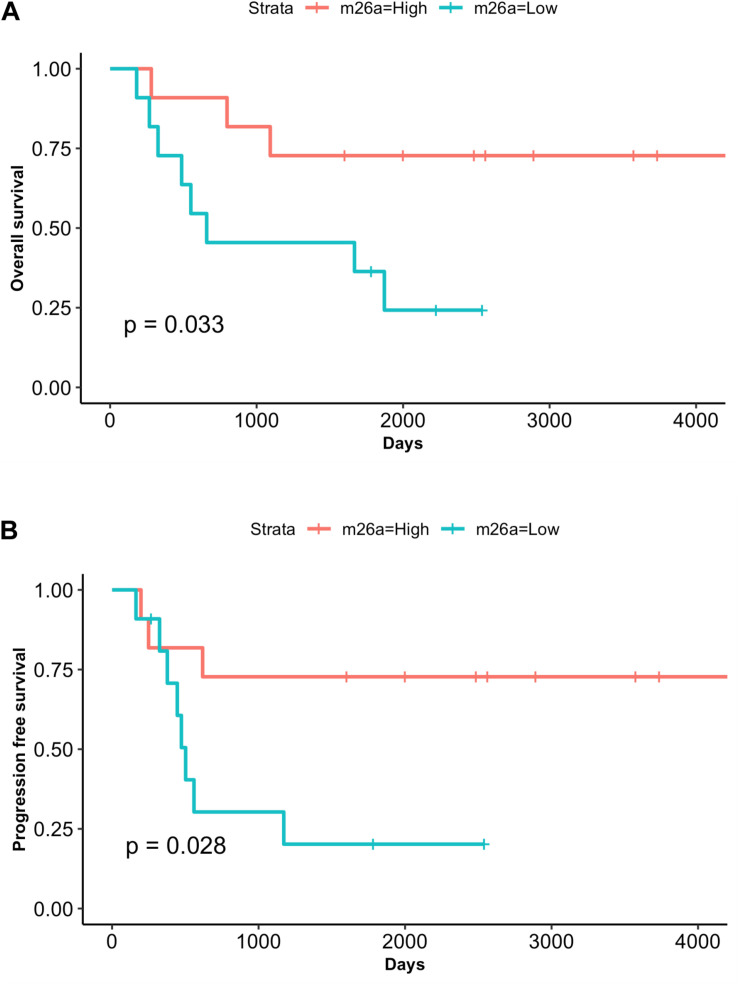
MiR-26a low plasma levels correlate with adverse outcome. Kaplan– Meier and log-rank analysis for overall survival (OS) **(A)** and progression-free survival (PFS) **(B)** were performed for the cohort of RMS patients (*n* = 22) based on low or high miR-26a plasma levels (median value). The group of patients with lower miR-26a plasma levels displays a significantly poor outcome (OS, *P* = 0.033; PFS, *P* = 0.028).

With the aim to test the prognostic value of miRNAs as well as the utility of measuring circulating miRNAs in plasma of RMS patients we performed survival analysis. Patients were divided according to median miRNA plasma levels. Remarkably, both overall (OS) and progression-free survival (PFS) analysis showed that patients low levels of miR-26a had poorer outcome compared to those with high miR-26a expression values, and this difference was statistically significant (OS *p* = 0.033; PFS *p* = 0.028, Figures 4A,B). Conversely, no survival correlation was found using miR-30b/30c median values (Supplementary Figures 2, 3). Univariate Cox regression analysis performed including clinical variables such as age, gender, fusion status, tumor size, IRS group confirmed these findings. According to it, circulating miR-26a was the only significant variable found to be associated with reduced OS and PFS in RMS patients (OS *p* = 0.047; PFS *p* = 0.041) (Supplementary Table 5).

## Discussion

In this study, we explored the utility of circulating miRNAs as biomarkers in pediatric rhabdomyosarcoma. The possibility to use a non-invasive approach such as a liquid biopsy to track a tumor-related marker at diagnosis and during follow-up is very attractive in pediatric solid tumors.

Rhabdomyosarcoma, an aggressive pediatric tumor with a mesenchymal origin, remains a challenge for patients that show a progressive disease or a relapse after treatment.

MiRNAs are largely studied in RMS, in particular the muscle-specific miRNAs, termed myomiRs, involved in the regulation of both cell proliferation and muscle differentiation, two key processes that control muscle development (Chen et al., 2006; McCarthy, 2008; Horak et al., 2016). Herein we report that, among myomiRs, circulating miR-206 results differentially expressed between patients and healthy donors, consistent with the observation that miR-206 is overexpressed in RMS primary tumor specimens as well (Missiaglia et al., 2017) and correlates with the presence of metastasis at diagnosis and worse overall survival of RMS patients (Missiaglia et al., 2010). Besides, only miR-206 plasma levels were found able at distinguishing RMS patients from non-RMS patients and healthy donors (Miyachi et al., 2010) unlike other common circulating RMS miRNAs, such as miR-1, miR133a and miR-133b.

On the contrary, miR26a/26b and miR30b/30c, which are miRNAs ubiquitously expressed in many cancers that act as tumor suppressors, were found to be downregulated in RMS patients’ plasma samples and somehow correlated to known negative prognostic factors and poor patient’s survival.

These findings were further supported by previously reported miRNAs expression data in RMS primary tumors, when compared with differentiated skeletal muscles and normal tissues (Missiaglia et al., 2017).

Indeed, low levels of miR-30b and/or miR-30c have been found in gastric cancer, renal carcinoma (Liu et al., 2017) and colorectal cancer (Zhao et al., 2019; Fan et al., 2020) with respect to control tissues. MiR30b/30c exert their tumor suppressor function by targeting genes involved in key processes of tumorigenesis, such as growth, survival, invasion and migration (Cao et al., 2017; Liu et al., 2017; Fan et al., 2020).

Likewise, microRNA-26 family members, miR-26a and miR-26b, have been found to be downregulated in various cancers respect to normal tissues. In esophageal tumor cells miR-26a/b were shown to be involved in a feedback loop with c-MYC that causes a decrease of proliferation (Li J. et al., 2017), whereas in hepatocellular carcinoma they acted as tumor suppressor sensitizing chemoresistant cancer cells to drug-induced apoptosis. Drug-induced autophagy is also impaired by miR-26a/b: this increases cancer cells susceptibility to drug exposure and promotes apoptosis (Jin et al., 2017). A recent study on multiple myeloma (MM) confirmed the role of miR-26a as tumor suppressor. In this study miR-26a was found to act by targeting CD38, a cell-surface glycoprotein involved in MM cells invasion and metastasis, resulting both in the induction of apoptosis *in vitro* and drug sensitization of MM tumors *in vivo* (Hu et al., 2020). In c-MYC-induced lymphoma cell lines, downregulation of miR-26a has been reported to come along with cMYC-induced EZH2 expression, a histone methyltransferase member of the Polycomb-group (PcG) family of epigenetic gene silencers implicated in neoplastic development (Sander et al., 2008). Similarly, in C2C12 myoblasts, the increased expression of miR-26a occurs alongside the induction of cell differentiation, expression of muscle-specific genes myoD1 and myogenin, and Ezh2 downregulation of Chung and Tellam (2008), whereas in rhabdomyosarcoma tumor cells miR-26a expression is lower and EZH2 is higher (Ciarapica et al., 2009). Our findings provide further evidence the key role of miR-26a in rhabdomyosarcoma cells, proposing also a novel potential role as blood biomarker. The prospective to use miRNAs as novel biomarkers in liquid biopsies of cancer patients has been explored by many groups in the last years, proving that circulating miRNA levels are different in cancer patients and healthy subjects, and positively correlate with the presence of metastasis at diagnosis and poor overall survival of the former (Imaoka et al., 2016; Li H.Y. et al., 2017; Guo et al., 2018; Mazza et al., 2020). With respect to this, the usefulness of droplets digital PCR for the quantification of circulating miRNAs has been demonstrated, both when used to compare circulating miRNAs levels between cases and controls (Mangolini et al., 2015), as well as when used to monitor the absolute levels of tumor-related miRNAs in plasma of patients with solid tumors (Zhao et al., 2018; Mazza et al., 2020).

Taken together our study supports the importance to explore the use of liquid biopsies in rhabdomyosarcoma pediatric patients managing, in particular the clinical utility of circulating miRNAs as novel diagnostic and prognostic biomarkers for this disease.

By using a qPCR Cancer Panel to screen circulating miRNAs we confirmed three miRNAs significantly downregulated in RMS patients’ plasma sample, one of which (miR-26a) also associated with known negative prognostic factors (RMS histology and fusion status) and significantly correlated with worst outcome. Although we are aware that a wider analysis on a larger cohort of patients is warranted, these findings disclose for the first time the potential to detect the absolute levels of circulating miRNAs in RMS patients, for both diagnostic and prognostic purposes. In this context, our data enforced the key role of miR-26a as both tumor suppressor and regulator of myogenesis in pediatric rhabdomyosarcoma.

## Data Availability Statement

The original contributions presented in the study are included in the article/Supplementary Material, further inquiries can be directed to the corresponding author.

## Ethics Statement

The studies involving human biological samples were reviewed and approved by the local Ethics Committee as part of a clinical trial currently carried out with the Associazione Italiana Ematologia Pediatrica (AIEOP = Italian Association of Pediatric Hematology and Oncology. The patients/participants provided their written informed consent to participate in this study.

## Author Contributions

LT conceptualized the study and wrote of the manuscript. CM and BP performed the experiments. AZ provided the biological samples. MC performed the statistical analysis. PB revised the manuscript. GB revised the manuscript, supervised the work, and funding acquisition. All authors reviewed and approved the final manuscript as submitted.

## Conflict of Interest

The authors declare that the research was conducted in the absence of any commercial or financial relationships that could be construed as a potential conflict of interest.

## Footnotes

1 https://software.broadinstitute.org/morpheus
