# Supplementary material for: Circulating miR-26a as Potential Prognostic Biomarkers in Pediatric Rhabdomyosarcoma
Source: Front Genet. 2020 Dec 10;11:606274. doi: 10.3389/fgene.2020.606274 (PMC7758343; doi:10.3389/fgene.2020.606274)
Supplement: Supplementary file 1 [file Data_Sheet_1.zip › Supplementary Table 5.PDF]

Clinical variable	Univariate p-value (OS)	Univariate p-value (PFS)
Gender	0.228	0.736
Age	0.539	0.900
Fusion	0.171	0.178
Size	0.727	0.645
IRS	0.097	0.370
miR-26a	**0.047**	**0.041**
miR-26b	0.150	0.127
miR-30b	0.150	0.127
miR-30c	0.698	0.611

Table S5. Univariate Cox regression analysis for overall survival and progression free survival. Univariate overall and progression-free survival analysis using Cox's regression model shows a significance only for plasma miR-26a levels, which are dichotomized depending on whether higher or lower than the median value. Since only miR-26a levels were significant in univariate analysis, multivariate analysis that considers the simultaneous effect of more covariates was not performed.
