# Supplementary material for: Circulating miR-26a as Potential Prognostic Biomarkers in Pediatric Rhabdomyosarcoma
Source: Front Genet. 2020 Dec 10;11:606274. doi: 10.3389/fgene.2020.606274 (PMC7758343; doi:10.3389/fgene.2020.606274)
Supplement: Supplementary file 2 [file Image_1.PDF]

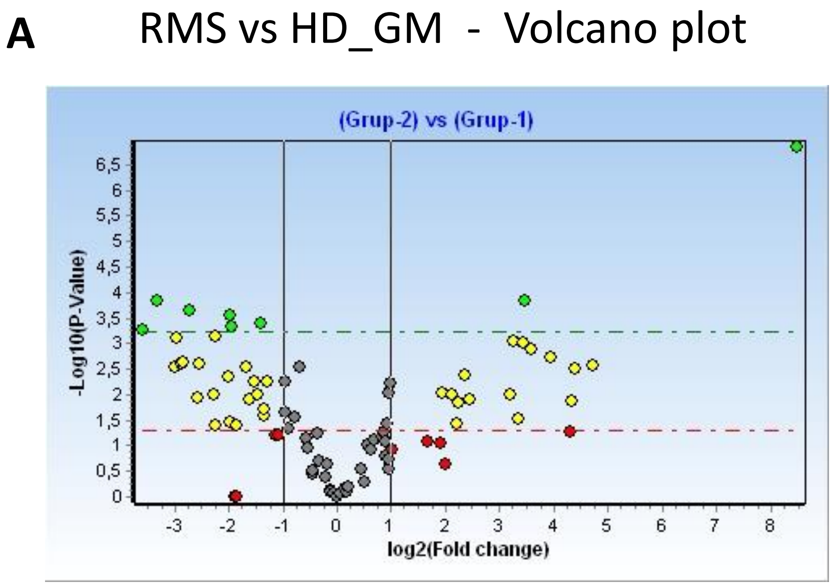

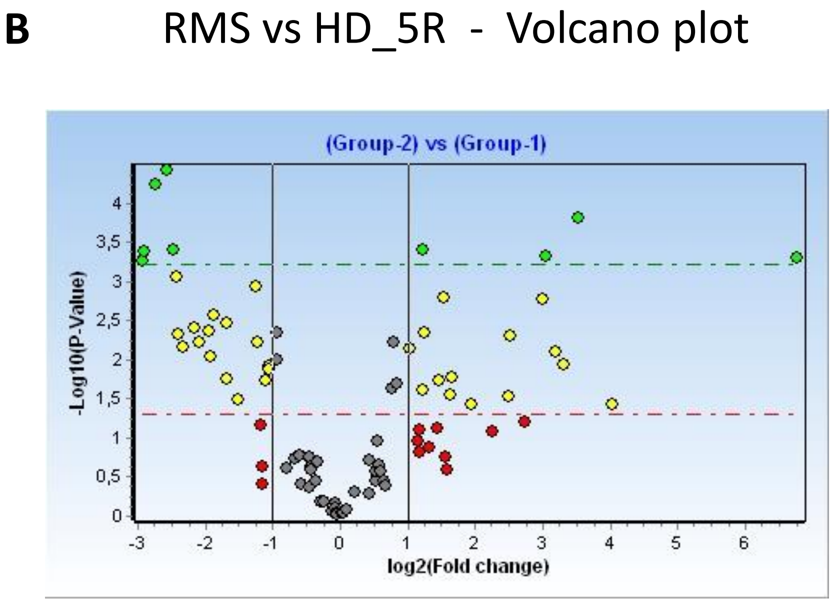

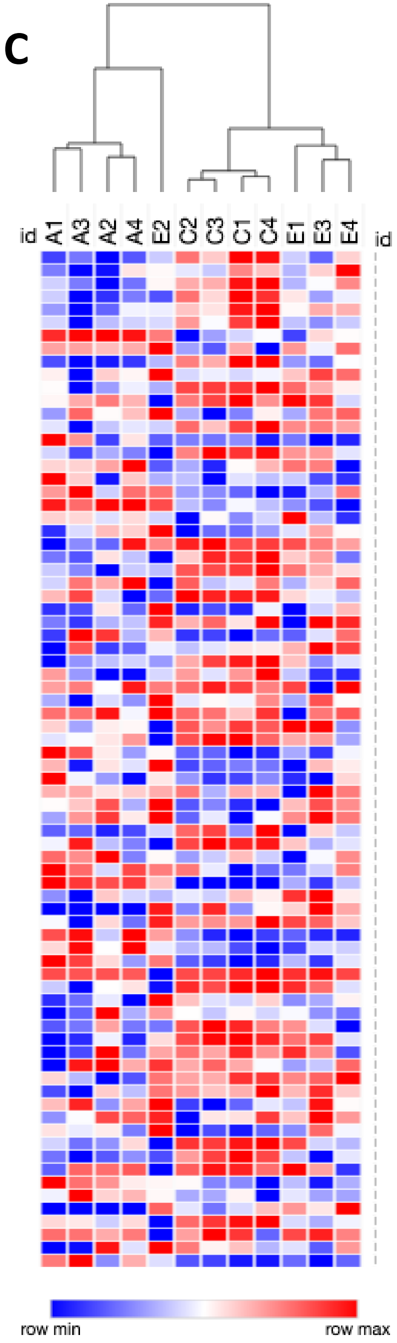

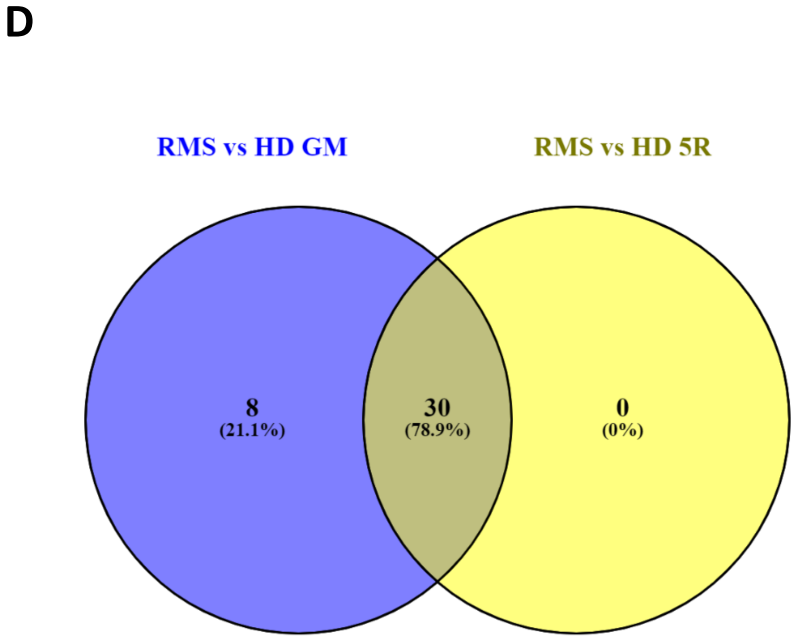

Figure S1. qPCR Cancer Panel data analysis. Volcano plots relative to global mean normalization (**A**) or to 5 reference genes analysis (**B**) considering RMS(Grup-2) versus HD(Grup-1). In (**C**) an unsupervised hierachical clustering was perfomed considering all 84-cancer associated miRNAs. In (**D**) the Venn analysis shown the good overlap of the data obtained with the two analysis: about 80% of differentially expressed miRNAs are common.
