# Supplementary figures and images for: Circulating miR-26a as Potential Prognostic Biomarkers in Pediatric Rhabdomyosarcoma
Source: Front Genet. 2020 Dec 10;11:606274. doi: 10.3389/fgene.2020.606274 (PMC7758343; doi:10.3389/fgene.2020.606274)

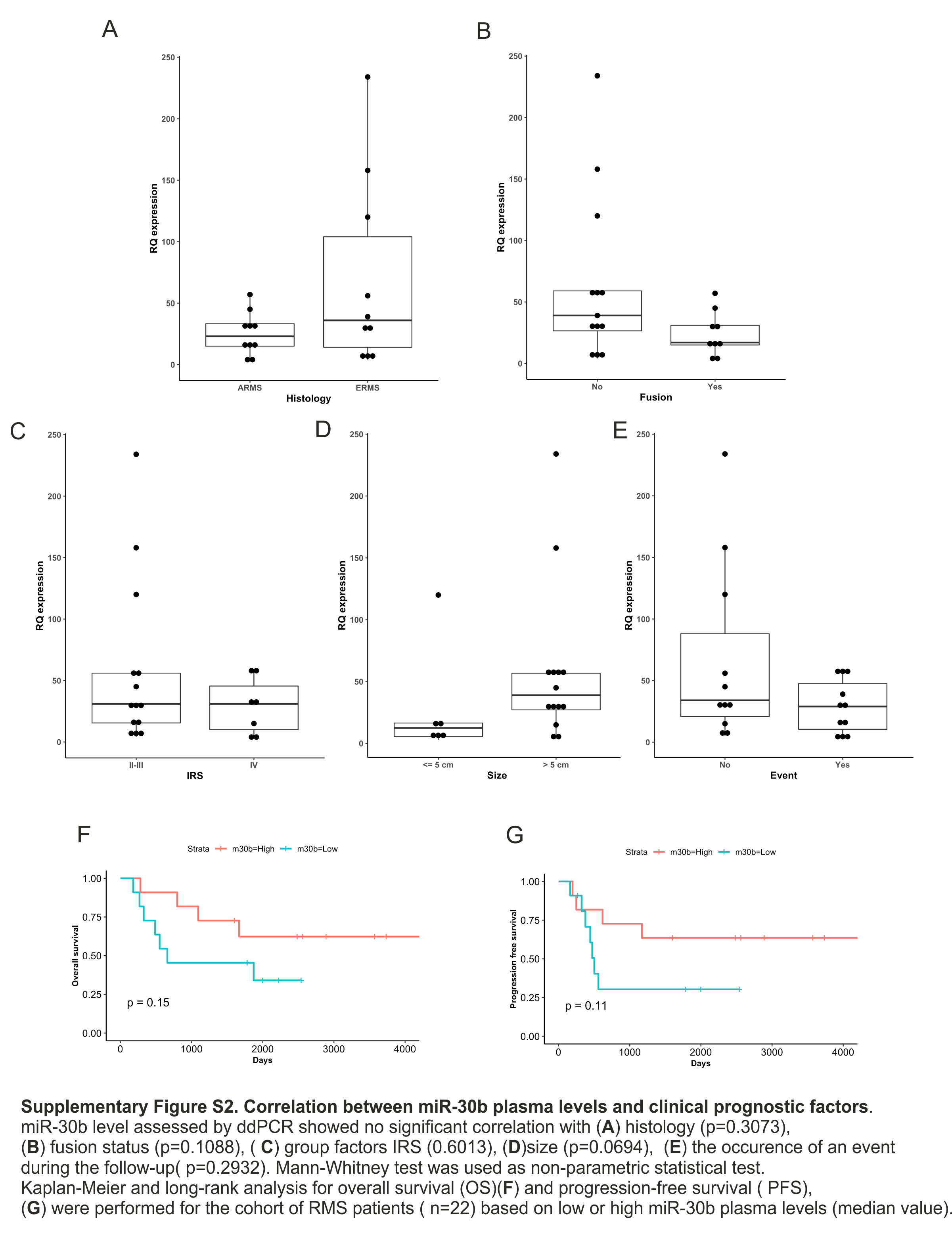

Supplement: Supplementary file 3 [file Image_2.TIF]

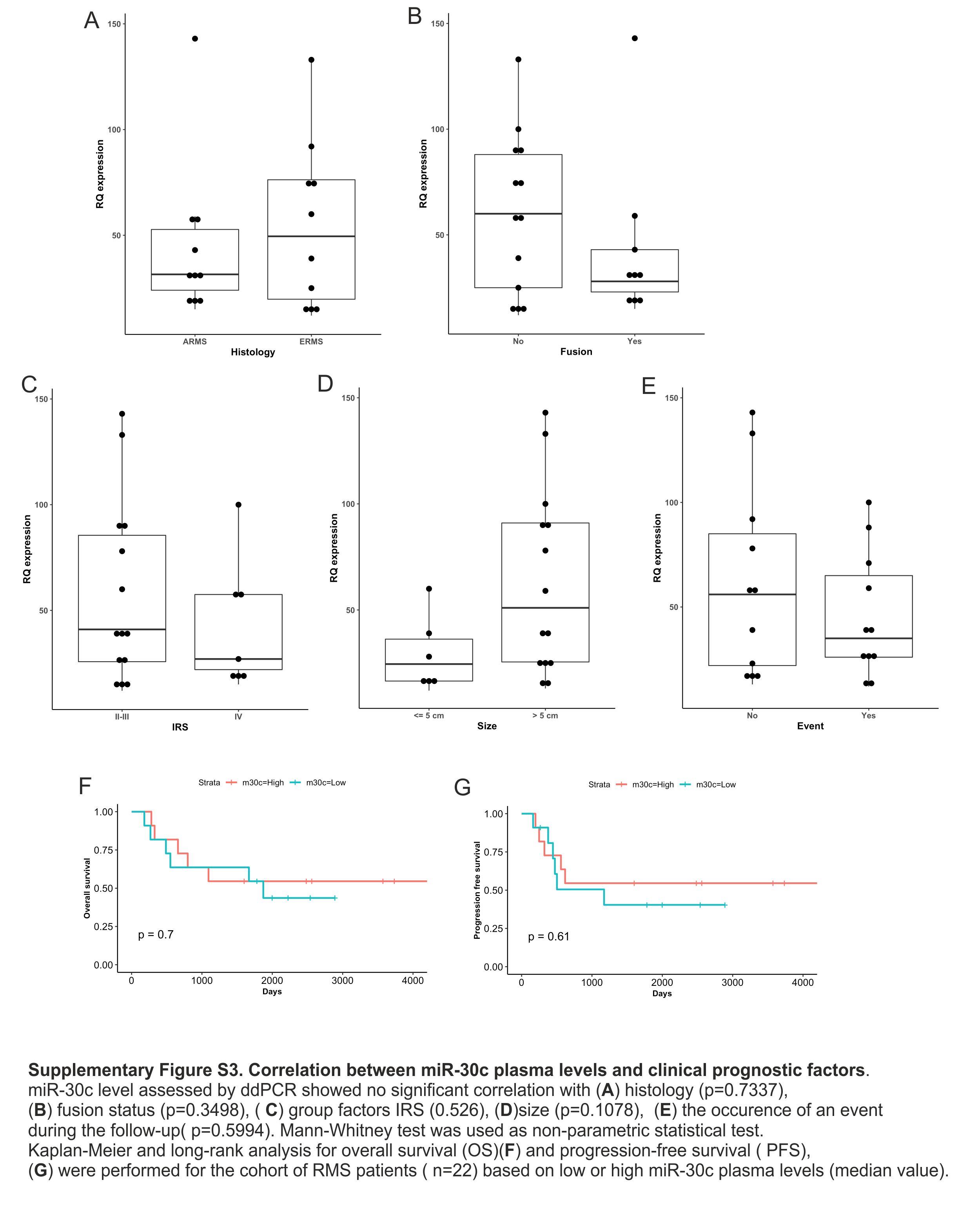

Supplement: Supplementary file 4 [file Image_3.TIF]
